# Development of a Targeted Multi-Disorder High-Throughput Sequencing Assay for the Effective Identification of Disease-Causing Variants

**DOI:** 10.1371/journal.pone.0133742

**Published:** 2015-07-27

**Authors:** Maria Delio, Kunjan Patel, Alex Maslov, Robert W. Marion, Thomas V. McDonald, Evan M. Cadoff, Aaron Golden, John M. Greally, Jan Vijg, Bernice Morrow, Cristina Montagna

**Affiliations:** 1 Department of Genetics, Albert Einstein College of Medicine, Bronx, New York, United States of America; 2 Divisions of Developmental Medicine & Genetics, Children's Hospital at Montefiore, Bronx, New York, United States of America; 3 Department of Medicine/Cardiology, Albert Einstein College of Medicine, Bronx, New York, United States of America; 4 Department of Pathology, Albert Einstein College of Medicine, Bronx, New York, United States of America; University of Texas MD Anderson Cancer Center, UNITED STATES

## Abstract

**Background:**

While next generation sequencing (NGS) is a useful tool for the identification of genetic variants to aid diagnosis and support therapy decision, high sequencing costs have limited its application within routine clinical care, especially in economically depressed areas. To investigate the utility of a multi-disease NGS based genetic test, we designed a custom sequencing assay targeting over thirty disease-associated areas including cardiac disorders, intellectual disabilities, hearing loss, collagenopathies, muscular dystrophy, Ashkenazi Jewish genetic disorders, and complex Mendelian disorders. We focused on these specific areas based on the interest of our collaborative clinical team, suggesting these diseases being the ones in need for the development of a sequencing-screening assay.

**Results:**

We targeted all coding, untranslated regions (UTR) and flanking intronic regions of 650 known disease-associated genes using the Roche-NimbleGen EZ SeqCapV3 capture system and sequenced on the Illumina HiSeq 2500 Rapid Run platform. Eight controls with known variants and one HapMap sample were first sequenced to assess the performance of the panel. Subsequently, as a proof of principle and to explore the possible utility of our test, we analyzed test disease subjects (n = 16). Eight had known Mendelian disorders and eight had complex pediatric diseases. In addition to assess whether copy number variation may be of utility as a companion assay relative to these specific disease areas, we used the Affymetrix Genome-Wide SNP Array 6.0 to analyze the same samples.

**Conclusion:**

We identified potentially disease-associated variants: 22 missense, 4 nonsense, 1 frameshift, and 1 splice variants (16 previously identified, 12 novel among dbSNP and 15 novel among NHLBI Exome Variant Server). We found multi-disease targeted high-throughput sequencing to be a cost efficient approach in detecting disease-associated variants to aid diagnosis.

## Introduction

Genetically heterogeneous disorders are challenging to diagnose due to the presence of multiple genes underlying their pathogenic mechanism [1–3]. High throughput next-generation sequencing (NGS) has the potential to improve clinical care by identifying genetic risk or actionable variants in a time-sensitive and cost-effective manner. In recent years, technological advances have made it possible to sequence entire exomes (whole exome sequencing, WES) or genomes (whole genome sequencing, WGS) with the final goal of better understanding the genetic underpinnings of a patient's disease. However, for diseases for which candidate genes are known, the use of a targeted approach has become a preferred method of choice in terms of clinical utility. This is because targeted sequencing reduces incidental findings, delivers higher sequence depth, allows more streamlined data analysis, and is lower in cost than WES and WGS due to pre-capture pooling and high multiplexing capabilities. However, the cost associated with genetic testing in clinical settings, even for targeted sequencing, is substantial when outsourced to commercial companies and becomes prohibitive for a low-income population, such as that of Bronx County, considering that the South Bronx where our hospital is located is the 2nd highest poverty ranked district throughout the US [4]. In addition patients with heterogeneous disorders, such as hypertrophic or dilated cardiomyopathies, that could be caused by alterations in more than 50 genes [5] may require, if carried on using commercial clinical tests, the use of 2–3 different sequencing panels to accurately diagnose the specific myopathy with an associated cost for each panel ranging from $1,500 to $4,000 [6].

In addition to economic status being a limiting factor for these types of genetic tests aimed to improve health care, there are limited variant data available reflecting the mixed population of Caribbean and Dominican Hispanic populations that make up the majority of the population in the Bronx. In this type of setting, this poses a challenge both in diagnosis and treatment [7–9].

To begin addressing these issues and to establish the potential clinical impact of a large target-sequencing panel, we developed a low-cost high-throughput multi-disease NGS assay for the analysis of patients seeking care in the Bronx. The assay targets 650 candidate genes associated with different disorders including rare pediatric Mendelian disorders, sensorineural hearing loss, abnormalities of cardiac rhythm, and cardiomyopathies. These disorders were chosen based on volume and clinical interest from our affiliated hospital in Bronx County (Montefiore Medical Center). The sequencing data were combined with Affymetrix Human Genome Wide SNP 6.0 Array data to identify potential pathogenic copy number variants (CNVs) in the same patients. We summarize the design process, bioinformatic pipeline and interpretation of results targeting a multi ethnic population from the Bronx presenting with various heterogeneous disorders. We provide the results of our analysis, highlight the difficulties we encountered throughout the process, and discuss our approach to overcome the main obstacles.

To our knowledge, this is the first report using targeted NGS to investigate multiple disorders in one comprehensive assay for the diagnosis of an underserved population. While the overall utility and success rate of NGS in challenging clinical settings require further analysis including the analysis of a larger patient cohort, we believe this study is fundamental as a starting point for implementing targeted NGS in our environment and other hospitals facing similar challenges.

## Materials and Methods

### Subjects

This study was approved by the Albert Einstein College of Medicine Committee on Clinical Investigations (CCI#99–201), a written informed consent for publication of this case report and any accompanying images was attained from each patient or guardian during patient enrollment. A copy of the written consent is available for review by the Editor of this journal. A total of 25 samples were included in this study. The DNA from eight patients with confirmed molecular aberrations (proof-of-principle cohort) were used to assess the performance of the custom panel and Affymetrix Genome-Wide SNP Array 6.0. Six samples had variants within genes included in our panel (*BCOR*, *CFTR*, *TRPC6*, *G6PC*, *HEXA*, *GBA*, *CIB2*) and two had CNVs (8p23.2 [2524538–3288481] x 3; 18q22.3 [71791767–71878869] x 3) that were previously detected through the use of a clinical microarray. In addition to patient samples, DNA from one HapMap lymphoblastiod cell line (NA12878) was obtained from Coriell Cell Repositories (Camden, NJ) and used to further test the sensitivity and specificity of our custom gene panel. Sample details and previously known variants can be viewed in Table 1.

**Table 1:** Proof of Principle Cohort: Samples with previously identified sequenced variants (samples 1–5) and previously identified copy number changes (samples 6–7).

Sample number	ID	Disorder	Gene	Validated mutation	Zygosity	Depth
1	TG322.001	Microphthalmia, syndromic 2	*BCOR*	c.4540C>T, p.R1480X	Het	1401
2	TG472.001	Focal Segmental Glomerulosclerosis	*TRPC6*	c.2409+7dup	Het	786
3	TG478.001	Cystic Fibrosis	*CFTR*	c.3120+1G>A *Positive carrier*	Het	642
4	AJ177	Glycogen Storage Disease	*G6PC*	c.247C>T, p.R83C	Het	1757
	AJ177	Tay-Sachs	*HEXA*	c.1274_1277dupTATC, p.Tyr427IlefsTer5	N/A	920
5	JS6.200	Hearing loss	*CIB2*	c.556C>T, p.Arg186Trp *Positive carrier*	Het	1396
6*	TG17.001	Multiple congenital anomalies	*FBXO15TIMM21* (interstitial)	arr 18q22.3 (71791767–71878869)x3	N/A	N/A
7*	TG33.001	Multiple congenital anomalies	*CSMD1*	arr 8p23.2 (2524538–3288481)x3	N/A	N/A

*Proof-of-principle samples 6 and 7 were included in the sequencing test cohort.

Whole blood was collected from sixteen patients that were used as the disease cohort: eight were patients seen at the Children’s Hospital at Montefiore Medical Center, NY (four unrelated and two pairs of siblings) and were diagnosed with congenital malformations and/or unusual clinical findings; eight patients had a clinical diagnosis of known Mendelian disorders (cardiomyopathies and arrhythmias). Demographic and clinical information for all patients are listed in Table 2.

**Table 2:** Demographic, clinical features, and sequencing results of the sixteen patients in test cohort.

Sample number	ID	Sex	Ethnicity	Clinical Information	Genetic tests and results performed (clinically) before enrollment in study	Results from Multi-Disease Target Panel	
						Gene	Protein Change
**1**	**TG17.001**	M	AA	DD DF	Chromosome microarray (18q22.3 interstitial duplication) Rett syndrome (*MECP2*)William syndrome, FISH for subtelomeric rearrangement Fragile X	None to report	None to report
**2**	**TG33.001**	F	N/A	MCA DD Chronic lung disease Osteopathia Striata with cranial sclerosis	Chromosome microarray (8p23.2 duplication maternally inherited) Xq11.2 deletion, X-inactivation 63:37) FISH for subtelomeric rearrangements FISH for VCFS, CHARGE (*CHD7*)	PAX6 *ZNF674 CFTR*	p.S213* (rs201251689) p.I341T no ExAC p.N1224K (rs371475225)
**3**	**TG90.001 (Twins of TG90.002)**	M	Cau/His	MCA FSGS Ticks and possible Tourette Syndrome Speech delay Fine motor delay Marfan Syndrome-like features	Cystic Fibrosis (*CFTR*)	FBN1	p.G1334D
**4**	**TG90.002 (Twins of TG90.001)**	M	Cau/His	MCA FSGS Ticks and possible Tourette Syndrome Speech delay Fine motor delay Marfan Syndrome-like features	Cystic Fibrosis (*CFTR*)	FBN1	p.G1334D
**5**	**TG251.001**	F	Cau	DD DFID Epilepsy Williams-Beuren Syndrome-like characteristics Moderate anxiety	Chromosome microarray	*NLGN4X GBP1*	p.Y463fs p.R106G (rs143163513)
**6**	**TG274.001**	M	His	VSD SD Bilateral cleft lip and cleft palate Duplicated renal collecting system	Chromosomes and chromosome microarray	*BBS1 NPHS2 CFTR SEMA3E*	p.Y121C p.L104F p.G576A (rs18000098) p.G190G
**7**	**TG290.001**	F	Cau/Isr	DD Noonan Syndrome-like characteristics Clinodactyly Brachydactyly Left inguinal hernias, Epicanthal folds Suborbital fullness	Hearing loss (*GJB2*, *GJB6*, *LOXHD1*, *CX26*, *CX30*) Noonan Spectrum Panel (*PTPN11*, *RAF1*, *SOS1*, *KRAS*, *HRAS*, *BRAF*, *MEK1*, *MEK2*, *NRAS*, *SHOC2*, *CBL*, *SPRED1*) Chromosomes and chromosome microarray	SPRED1 NLGN4X	p.N10K (rs201692618) p.R101*
**8**	**TG292.001**	M	AA	PDD Tall stature Mild dysmorphism Macrocephaly Hypertelorism Flat nasal bridge	Fragile X Chromosome microarray	NXF5	p.R320* (rs140252282)
**9**	**TG471.001 (sister of TG471.002)**	F	His	DCM	None	*SCN5A LDB3 AKAP9 TMEM43*	p.R340Q (rs191009474) p.V118M (rs35507268) p.T3469M p.R220C
**10**	**TG471.002 (sister of TG471.001)**	F	His	DCM	None	*SCN5A VCL AKAP9 LDB3*	p.A672T (rs199473140) p.F212S p.T3469M p.V118M (rs35507268)
**11**	**CMB1**	F	His	Brugada Syndrome LQTS	None	*LDB3*	p.D232N (rs121908338)
**12**	**RB1**	F	Ben	CPVT	None	*CASQ2*	p.R67* c.532+1G>A
**13**	**AJ1**	F	Cau	SPOH	None	*None to report*	*None to report*
**14**	**JFM1**	F	AA	HCM	None	*PCSK9*	p.R469W (rs141502002)
**15**	**SS1**	F	AA	DCM with conduction disease	None	*SCN2A*	p.I1583T (rs367566833)
**16**	**MS1**	M	Mex	Brugada Syndrome	None	None to report	None to report

Legend: Ethnicitiy: AA = African American, Cau = Caucasian, His = Hispanic, Isr = Israeli, Ben = Bengali, Mex = Mexican, N/A = Not Available; Clinical Information: DD = Developmental Delay, DF = Dyspmorphic Features, MCA = Multiple Congenital Anomalies, FSGS = Focal segmental glomerulosclerosis, ID = Intellectual Disability, VSD = Ventricular Septal Defect, SD = Speech Delay, PDD = Pervasive Developmental Delay, DCM = Dilated Cardiomyopathy, LQTS = Long QT Syndrome, CPVT = Catecholaminergic Polymorphic Ventricular Tachycardia, SPOH = Severe Postural Orthostatic Hypertension, HCM = Hypertrophic Cardiomyopathy.

Two samples (TG478.001 and SS1) had been previously analyzed using whole exome sequencing (WES) and were included as an additional quality control (S1 Table).

### Sample preparation

DNA from all samples was isolated in the Molecular Cytogenetics Core at the Albert Einstein College of Medicine, NY according to standard protocols using the Puregene Genomic DNA Purification kit (Gentra, Minneapolis, MN). The quantity of the DNA was assessed by spectrofluorometer quantification using Quant-iT PicoGreen dsDNA Assay Kit, according to standard protocols (Quant-iT PicoGreen dsDNA Reagent and Kits, Molecular Probes, 2008. Invitrogen). Additionally, all samples were run on a 1% Agarose-TBE Blend gel to visualize any impurities.

### Capture design

**Fig 1:**
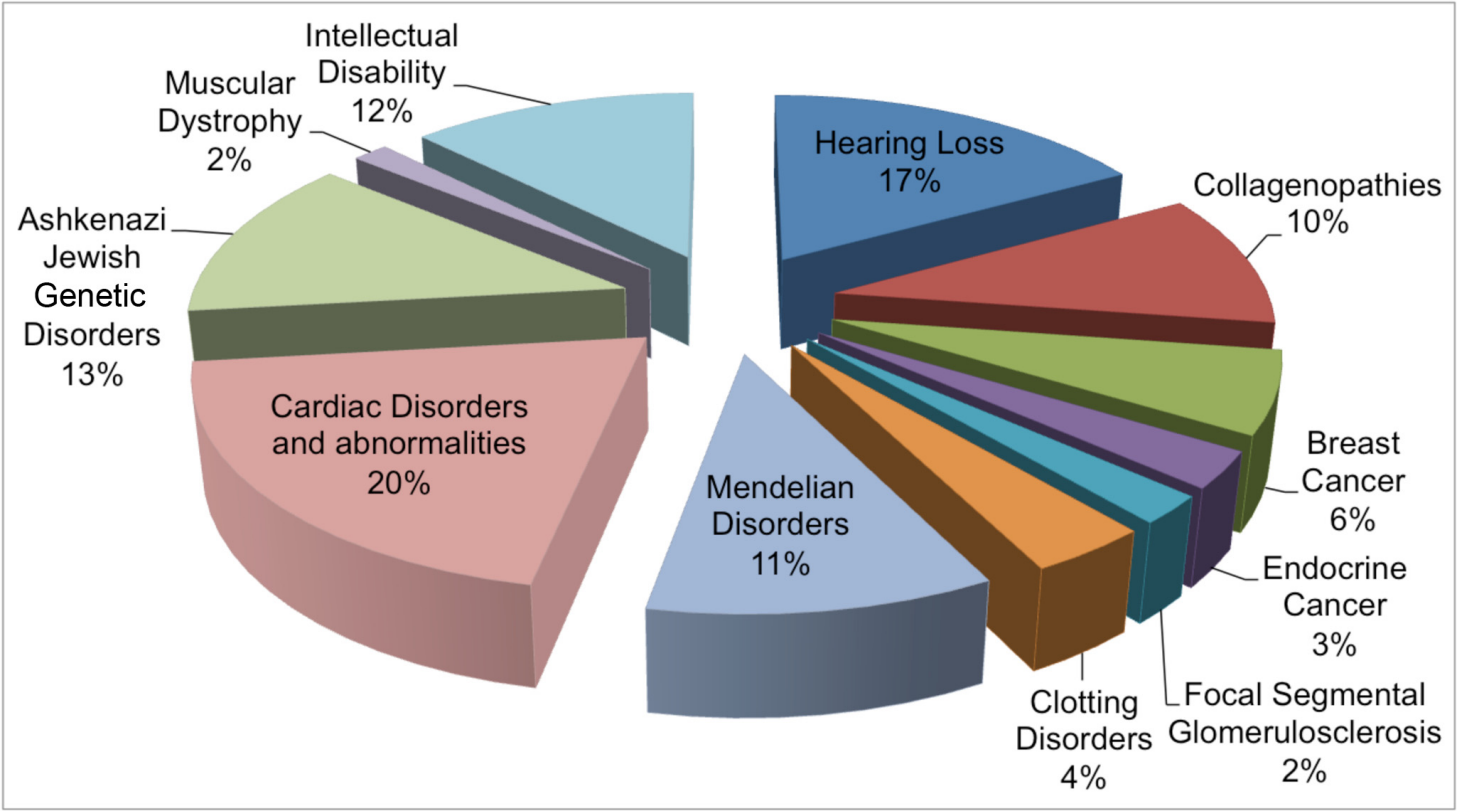
Custom panel design. The pie chart illustrates the percent of genes included in the custom design categorized based on specific diseases/abnormalities. Of note the Ashkenazi Jews variant disorders have been kept separate because they represent an ethnic division commonly associated with specific disease and genetic variants.

We designed a hybridization-based, multi-disease gene panel using Roche-NimbleGen's SeqCap EZ Choice Library capture system, which uses Sequence Search and Alignment by Hashing Algorithm (SSAHA) to select unique probes (Roche/NimbleGen, Madison, WI). In this custom design we targeted all exons, including 50 bp of the flanking intronic sequence and 1 kb areas upstream and downstream of each gene. Disease areas of interest were largely based from the outcome of an extensive investigative process involving all clinicians at our associated teaching hospital that were interested in genomic sequencing and who felt that their patients would benefit from NGS but were unable to afford it. Gene selection was based our discussions with collaborators Montefiore's clinicians, and the genes potentially related to their patients cohort selected based on current scientific knowledge. We also included genes that harbored previously reported variants for the samples chosen as a proof of principle validation. The custom panel captured 4.98 Mb of genomic sequence comprising 11,496 exons spanning 650 genes (complete gene list and associated syndromes are provided in S2 and S3 Tables). The percent breakdown of genes related to each disease and abnormality is summarized in Fig 1.

### Target Capture Sequencing Method

Libraries were prepared using 5 μg of genomic DNA according to the Illumina TrueSeq sample preparation protocol as described at http://support.illumina.com/content/dam/illumina-support/documents/myillumina/f5f619d3-2c4c-489b-80a3-e0414baa4e89/truseq_dna_sampleprep_guide_15026486_c.pdf (Illumina, San Diego, CA). After appropriate quality controls, libraries were pooled and captured using the SeqCap EZ Choice Library (Roche/NimbleGen, Madison, WI) following the manufacturer’s protocol, as detailed at the following http://www.nimblegen.com/products/lit/06560881001_SeqCapEZLibraryLR_Guide_v2p0.pdf. Captured libraries were multiplexed two-seven samples per lane and sequenced to generate 2x150 bp paired-end reads using the HiSeq2500 rapid run mode (Illumina, San Diego, CA).

### Whole Exome Sequencing

To establish sensitivity and specificity of our assay Whole Exome Sequencing (WES) libraries from samples SS1 and TG478.001 were prepared using 5 μg of genomic DNA according to the Illumina TrueSeq sample preparation protocol (Illumina, San Diego, CA). Each library was individually captured using SeqCap EZ Exome plus UTR Library (Roche/NimbleGen, Madison, WI) following the manufacturer's protocol. Captured libraries were sequenced individually to generate 2x150 bp paired-end reads using the Illumina HiSeq 2500.

### Copy Number Variation

All samples were submitted to Einstein's Genomics Core Facility to test for copy number variations (CNV). All samples were diluted to 10 ng/μL and analyzed by on Affymetrix Genome-Wide Human SNP Array 6.0 arrays following the manufacturer’s protocol (Affymetrix, Inc.).

### Bioinformatic pipeline

**Fig 2:**
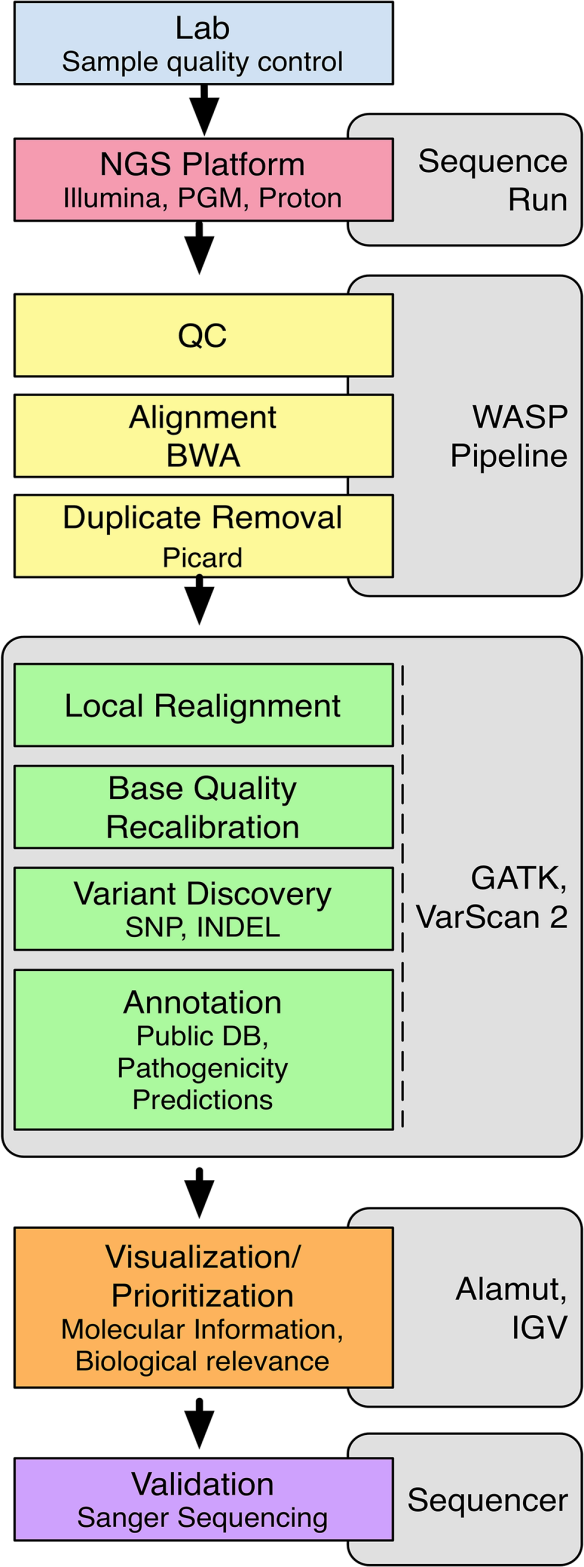
Project pipeline. Sample quality is conducted in the laboratory (blue box), subsequent library prep and sequencing is conducted in the Epigenomics Shared Facility (pink box). The sequence reads automatically progress into the WASP pipeline for quality control parameters, alignment to reference sequence through BWA and duplicate removal through Picard is performed (yellow boxes). Local Realignment, base quality recalibration, variant discovery and annotation take place via GATK or VarScan2 (somatic) (green boxes). Clinically relevant variants are prioritized through Alamut and visualized through IGV (orange boxes). Validation is then performed by Sanger sequencing and results are visualized through Sequencher 4.0.1 (purple box).

A comprehensive flowchart illustrating the pipeline used from sample QC through validation is shown in Fig 2. Samples were submitted through Einstein's Wiki-Based Automated Sequence Processor (WASP) system software [10] where sequencing reads were aligned and mapped to the human reference genome (GRCh37, UCSC hg19) using the Burrows-Wheeler Aligner (BWA) [11,12] algorithm, with removal of PCR duplicates using Picard [13]. Local re-alignment, base quality recalibration and variant calling were performed using the Genome Analysis Toolkit (GATK) version 2.0 [14] (Broad Institute, Cambridge, MA) applying the following quality parameters: sequencing depth ≥15X, base quality score ≥17, and mapping quality of ≥20. Variants that did not meet these quality standards were removed. We also removed sequence reads exceeding 10 bp from either exonic boundary, as we did not consider variants in this region interpretable due to lack of functional information.

**Fig 3:**
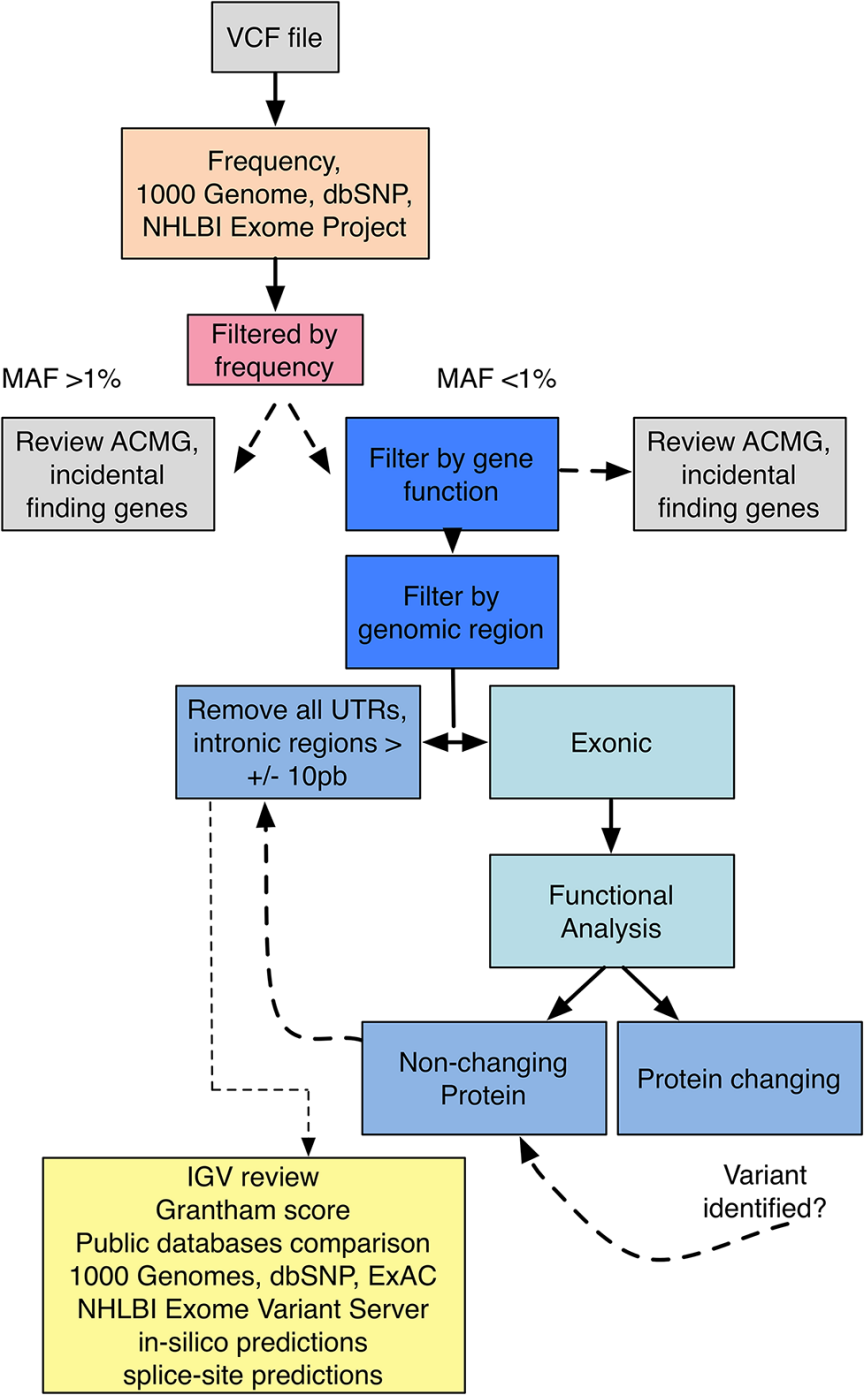
Variant Calling Pipeline. The vcf file created through the project quality control pipeline is filtered for frequency using 1000Genome, dbSNP, ExAC, and NHLBI Exome Project. All variants with a MAF <1% are then prioritized by gene function and genomic region. Variants associated with the patient's phenotype mapping to exons are further prioritized based on functional annotation. If protein-changing variants are identified in a gene of interest, further analysis of non-protein changing variants, UTRs, and intronic regions are analyzed to consider the possibility of compound heterozygous variants. Variants are visualized and reviewed though IGV and *In silico* prediction tools, and taken into consideration to estimate the possible impact of each variant at the protein level. Variants are also manually investigated through Alamut v2.2 software (Interactive Biosoftware, San Diego, CA) to aid in variant interpretation and pathogenicity predictions. Of note, we follow the ACMG guidelines for incidental findings (grey boxes).

Basic functional information (gene ID, genomic region, *etc*.) of the detected variants was achieved using ANNOVAR [15]. The gene set recommended by the ACMG for incidental findings was manually analyzed (Fig 3). We prioritized the remaining variants by excluding those with a minor allele frequency (MAF) >1% based on population allele frequency (dbSNP, 1000 Genomes Project, Exome Aggregation Consortium-ExAC and NHLBI Exome Project) [9,16,17] to assess only rare, potentially pathogenic variants. In addition, all synonymous variants regardless of MAF were removed from the analysis unless additional protein-coding variants were identified in the same gene (this filtering method was used to consider possible compound heterozygotes and recessive modes of inheritance). *In silico* prediction tools based on multiple factors such as Grantham score, conservation between species and functionality of domain harboring the variant were taken into consideration to estimate the impact of each variant at the protein level. Remaining variants including missense, nonsense, frameshift, indel and splice site junction (+10 bp) variants were manually investigated through Alamut v2.2 software (Interactive Biosoftware, San Diego, CA) to aid in variant interpretation and pathogenicity predictions. All variants of interest were visually inspected using the Integrative Genomics Viewer [18] (IGV) to assess the quality of variant calls and sequencing uniformity.

### Copy-number variation detection

Affymetrix Genome-Wide Human SNP Array 6.0 CN.CHP files that passed the recommended values for quality contrast (QC>0.4) and mapping (MAPD<0.3; Median Absolute Pairwise Difference) were analyzed and visually inspected through Affymetrix Chromosome Analysis Suite Software (ChAS). Copy number variants (CNVs) were called if they exceeded 200 kb, with at least 50 probes covering the region. Results have been reported based on the International System for Human Cytogenetic Nomenclature, ISCN 2013 (i.e. x 3 indicates a duplication at that particular locus).

### Validation

All variants of interest (see S4 Table) were PCR-amplified, purified and validated by Sanger sequencing (Genomics Core, Albert Einstein College of Medicine, NY). Primers were designed through Primer3 [19]. Results were visualized through Sequencher v4.0.1 software (Gene Codes, Ann Arbor, MI). Quantitative PCR was performed to validate any CNVs of interest. We designed primers amplifying 75-150bp spanning the CNV of interest. Control DNA (Human control genomic DNA, Promega) was used as a reference in all reactions. Test samples and controls were amplified using the Fast SYBR Green Master Mix on the Spot One Real Time PCR system (Life Technologies). Ct values of the samples were analyzed using the comparative Ct method also known as the 2–[delta][delta]Ct method, where [delta][delta]Ct = [delta]Ct,sample—[delta]Ct,control.

## Results

### Performance of the custom multi disease panel

#### Sequencing Statistics

We observed, per sample average, a mean depth of 540x, ranging from minimal 260x to maximal 1230x (Tables 3 and S5). Coverage varied per sample across the same target, which is a known issue previously reported in custom targeted-capture assays [5,20]. On average, 81% of all targeted bases were covered by at least 20 reads (minimum 73% to a maximum of 91%) and 80% aligned to the reference sequence (70%- 87%), which was similar between proof-of-principle and test samples. Studies suggest that high GC content or repetitive elements could be responsible for poorly covered regions [20,21]. Therefore GC% for each exon was calculated through Einstein's Computation Core from our target file and compared the mean depth of each exon for each sample. Strong correlation between GC-content and poorly covered regions was not identified. Studies suggest that probe optimization, library automation, and minor changes to the library preparation protocol can increase uniformity and reduce sample-to-sample variability [22].

**Table 3:** Summary of sequencing coverage and detected variants for test cohort.

Samples	Total Reads	Avg. Depth (X)	% Bases at ≥ 20X coverage	Total no. of SNVs	Total no. of SNVs at ≤ 1% MAF	Total no. of Indels	Total no. of Indels at ≤ 1% MAF
TG17.001*	48581252	496	81	4306	402	790	458
TG90.001*	46810778	498	79	3593	186	674	313
TG90.002*	61418574	582	80	3616	186	652	312
TG251.001*	41042110	448	77	3517	131	662	321
**TG274.001**	53239778	546	88	3662	205	685	367
**TG290.001**	47030442	514	88	3670	189	685	375
**TG292.001**	47609338	491	88	4481	751	776	386
**TG471.001**	44839312	416	89	3630	245	646	310
**CMB1**	37422180	263	86	3905	223	691	348
AJ1*	68089872	627	78	3323	150	643	304
RB1*	39783474	298	73	3354	135	648	304
JFM1*	40765488	446	75	4075	328	784	371
SS1*	60697664	533	77	4093	363	758	371
MS1*	53780084	327	73	3298	140	626	284
TG33.001*	42699994	417	75	3310	154	667	312
**TG471.002**	127605702	1217	91	3787	255	696	322
**Average:**		**460**	**80**	**3722**	**252**	**693**	**342**

* indicates samples that were multiplexed together.

TG471.002 was added to another lane for logistic reasons.

#### Evaluating panel performance using samples with known variants

Performance of the panel was evaluated by Roche-NimbleGen and carried-out through comparison of variants detected in HapMap sample NA12878 against those identified in NA12878 analyzed independently as part of the 1,000 Genomes Project data set [17]. Within the regions targeted by our panel, a total of 1946 true positive SNPs were detected, yielding 97.60% sensitivity. SNP classification revealed 15 misclassified genotypes out of the 1946 detected variants, yielding 99.20% specificity.

Additionally, of all the samples used in this study, five had known variants previously identified as part of a clinical follow-up and were used as a proof of principle (Table 1, samples 1–5). The known variants ranged from missense, nonsense, frameshift, and indels. All six variants were detected in their correct allelic state.

#### Identification and prioritization of disease-causing variants

Through the pre-pool custom capture and multiplexing approach, we were able to detect an average of 3,726 SNVs and 693 indels per sample. A custom variant filtering strategy (minor allelic frequency <1%, functional information, in silico pathogenicity predictions; see methods and Fig 3) was applied to identify rare, possibly pathogenic variants in each of the samples. This strategy produced an average of 252 candidate SNVs and 342 candidate indels per sample (Table 3). We subsequently prioritized these variants by assessing gene function in concordance with the observed phenotype. This ultimately resulted in a curated list of a total of 22 missense, 4 nonsense, 1 frameshift, and 1 splice variants (16 previously identified, 12 novel among dbSNP and 15 novel among NHLBI Exome Variant Server) relevant to the phenotype for all sixteen samples.

### Variant Identification for samples with complex pediatric diseases

The cohort analyzed in this study includes patients with various pediatric disorders. Some are considered Mendelian disorders that have genetic heterogeneous origins, while others are complex disorders with less well known etiologies [23]. Variance between gene-environment and gene-gene interactions can be responsible for complex inheritance and lack of penetrance causing discordant phenotypes observed in relatives within the same family, making treatment, diagnosis and risk recurrence particularly difficult to assess [23,24].

Eight patients presented with multiple congenital anomalies, mental illness and craniofacial disorders. All variants and patient clinical features are summarized in Table 2. These variants should be ultimately tested for altered gene functions and for effects on the protein to validate that they are pathogenic and to establish their contribution to altered gene function. Below, we highlight two cases from this cohort in which we identified variants with potential diagnostic value.

#### CASES 1 and 2

Two fourteen-year-old male siblings (twins) TG90.001 and TG90.002, were initially referred to the Montefiore Pediatric Genetics clinic for evaluation of multiple congenital anomalies. At age four, both boys were diagnosed with focal segmental glomerulosclerosis (FSGS) by renal biopsy and were also noted to have tics, which were felt by their neurologist to be due to a *forme fruste* of Tourette syndrome. Developmentally, motor and cognitive milestones were slightly delayed, and both had dolichostenomelia with arm spans significantly greater than height. In addition, each had a high arched palate, joint laxity, pectus excavatum, mild scoliosis, and pes planus (flat feet). All of these physical findings suggested a diagnosis of Marfan syndrome (MS) (OMIM#154700). However, since the urologic, renal and developmental problems affecting the siblings are not typically seen in MS, and cardiac evaluations were normal, it was unclear which testing should be performed. At the time of this evaluation, *Fibrillin 1* (*FBN1)* testing was not clinically available. Testing through our panel revealed that both patients carried one previously-reported missense variant in the coding region of the *FBN1* gene (c.G4001A; p.G1334D; rs191989961), which produces a guanine-aspartate change and it is predicted as probably-pathogenic by our *in silico* analysis. This variant was previously reported in one other subject by the Laboratory for Molecular Medicine (Cambridge, MA) and deemed likely pathogenic based on their systematic approach for variant classification, which considers disease association from existing data, mechanism of gene function, and the use of several bioinformatic tools to predict the possible impact of the variant on the gene [25]. MS is inherited in an autosomal dominant manner and since variants in *FBN1* are also commonly associated with MS, we believe our findings may help explaining the boys’ MS-like features. There were no variants identified in the FSGS-associated genes present in our panel, suggesting that additional testing is needed in these patients to determine the underlying molecular cause for their renal and urologic phenotype. Of note, if testing for these patients were to be performed clinically by Sanger sequencing or using a disease-specific gene panel, the costs associated would have been greater given that two separate testing for each phenotype would have been required.

#### Patients with Mendelian cardiomyopathies and arrhythmias

The cardiac cohort included patients with heritable and potentially lethal disorders of cardiac rhythm followed in the Montefiore-Einstein Center for CardioGenetics. This group was comprised of individuals with cardiomyopathies and channelopathies, including hypertrophic cardiomyopathy (HCM, OMIM#192600, 1 patient), dilated cardiomyopathy (DCM; OMIM#115200; 3 patients, two siblings, one unrelated), Brugada syndrome (BS; OMIM#601144, 2 unrelated patients), long QT syndrome (LQTS; OMIM#192500, same patient with Brugada syndrome), and catecholaminergic polymorphic ventricular tachycardia (CPVT; OMIM#604772, 1 patient). In the eight patients tested (2 related; 6 unrelated), we identified 15 rare variants mapping to 9 of the 154 cardiac specific genes included in our panel (Table 2). We were able to provide an accurate diagnosis for one patient to be discussed below.

#### CASE 3

A 29-year-old woman, RB1, was referred to the Montefiore Center for CardioGenetics for evaluation. She had experienced episodes of palpitations and syncope related to exercise since 11 years of age. Due to intermittent prolongation of her QT interval on electrocardiography (ECG), she was given a provisional diagnosis of either Long QT syndrome (LQTS) or CPVT. These disorders have some overlapping features and both, if left untreated, may result in sudden death. However, because the treatment for various forms of LQTS and CPVT differ, determining the exact genetic variants is extremely helpful in managing patients. In addition, identifying the genetic variant can be life-saving for first degree relatives. Commercial testing is available for this specific phenotype but requires two separate sequencing panels (one for LQTS and one for CPVT) to differentiate the diagnosis. Because of lack of insurance and high cost, commercial testing was never performed. Based on the custom multi-disease gene panel that we designed, this patient was identified to be a compound heterozygote for variants in the *calsequestrin 2* (*CASQ2)* gene, identifying one novel stop variant (c.C199T, p.Gln67*) and one novel splice site variant (c.532+1G>A). Sanger sequencing confirmed the variants and in addition detected the presence of the p.Gln67* variant in the patient's child. The splice site variant was absent in the child, indicating that the mother's variants were *in trans*. Variants in *CASQ2* account for 5–7% of all cases of CPVT, suggesting the most likely mode of inheritance to be autosomal recessive. Although the c.532+1G>A variant has not yet been demonstrated to alter mRNA splicing, we are conducting further functional work to determine how this variant affects splicing.

#### CASES 4 and 5

**Fig 4:**
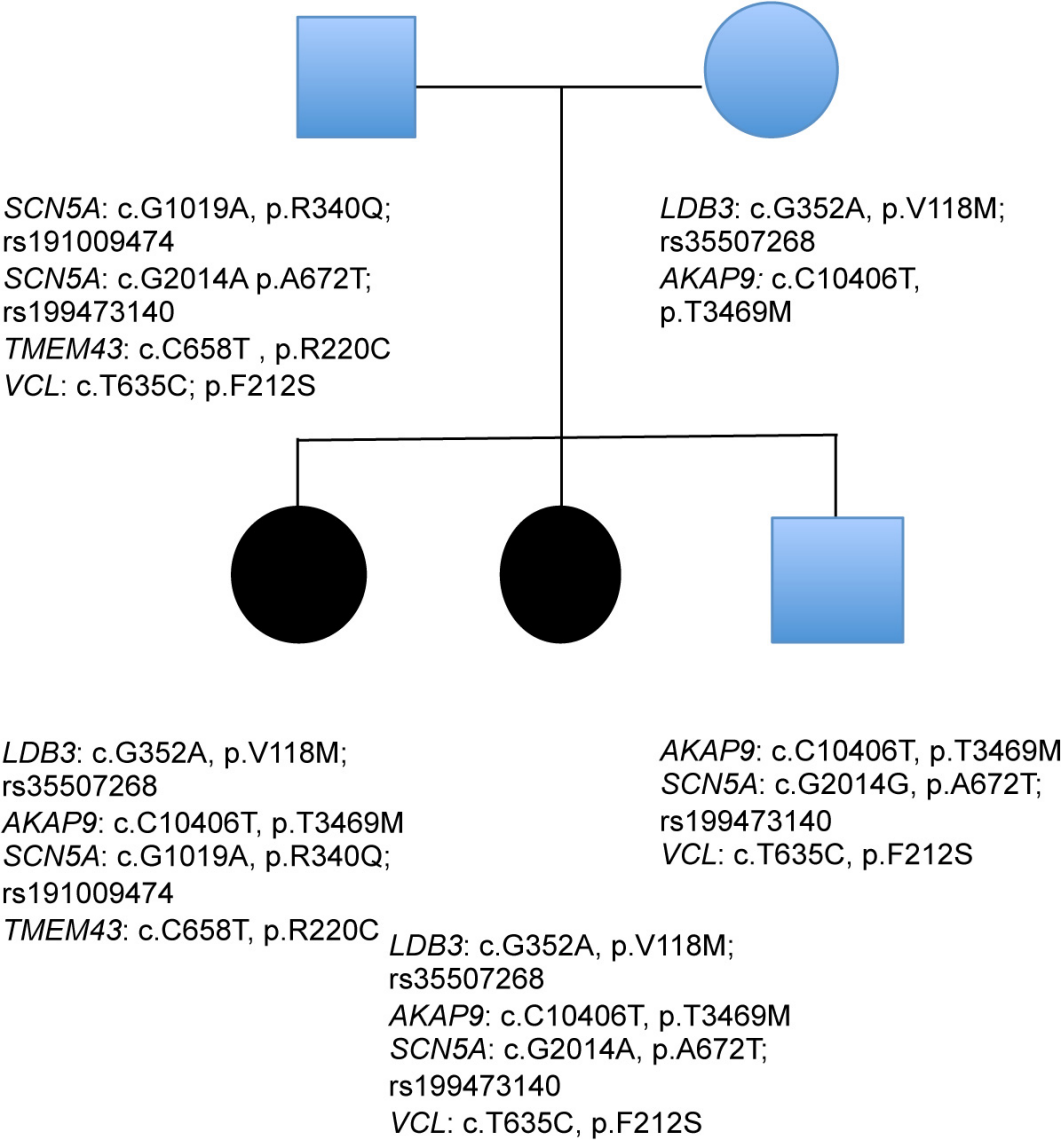
Mutational pedigree of TG471.001 and TG472.002, sisters with DCM. Affected sisters are indicated in black. Associated variants are provided under each family member.

The DNA from two sisters, TG471.001, TG471.002, presented to the Center for CardioGenetics with overlapping phenotypes of Dilated Cardiomyopathy (DCM), were sequenced and all cardiac related genes were analyzed. Several variants associated with cardiac disorders were detected, but only the *LDB3* gene is directly associated with DCM (*LIM domain-binding 3* gene *LDB3;* Cardiomyopathy, Dilated, 1C; OMIM#601493) and both sisters harbored the same missense variant (c.G352A, p.V118M; rs35507268). However, this exact *LDB3* variant was also observed in the unaffected mother and upon further investigation categorized as likely benign by dbSNP, ClinVar, and HGMD. The other variants identified in the sisters were also identified in the unaffected family members, suggesting a lack of a clear pathogenic effect (see Fig 4 for mutational pedigree). Based upon this analysis, the true disease causing variants remain to be identified. We recommend whole exome sequencing be performed on this family in order to identify additional variants potentially associated with DCM. CNV analysis performed on the sisters did not identify variations suggestive of a potentially pathogenic nature.

### Copy number variation results

In the 18 samples analyzed, a total of 152 copy number deletions and 351 copy number amplifications were detected. All copy number changes highlighted below may have potential diagnostic value and were validated through qPCR (S1 Fig and S4 Table).

#### CASE 6

Patient SS1, presenting with a dilated cardiomyopathy (DCM) with conduction defects (OMIM#115200) harbored a 3.74 kb deletion that resulted in the heterozygous loss of the *alpha-T-catenin* gene (*CTNNA3*). This gene maps to chromosome 10q21.3, a region linked to autosomal dominant familial DCM [26]. The most common form of myocardial disease, DCM is associated with a high incidence of ventricular arrhythmias, severe congestive heart failure and an increased risk of sudden death [27]. *CTNNA3* is highly expressed at intercalated discs of cardiomyocytes and peritubular myoid cells of the testis, indicating a specific function for this gene in particular muscle tissues [26]. Several reports link variants in the *CTNNA3* gene with another form of cardiomyopathy, specifically, arrhythmogenic right ventricular dysplasia [28] (ARVC; OMIM#107970). Li *et al*. examined the role of *CTNNA3* in the hearts of mice and found that CTNNA3 acts as a molecular integrator of adherens junctional and desmosomal components at the area composita resulting in early onset of DCM [27]. This CNV is not common and to our knowledge has never been reported based on a literature search or inquiry of CNV databases [29]. We propose the deletion may be causative of the patient's phenotype.

Patients TG90.001 and TG90.002 (CASES 1 and 2 above), both harbored two duplications mapping to regions 2q21.1 and 6q27, resulting in an allelic gain to eleven and four genes, respectively. Two genes, *tubulin*, *alpha 3d* (*TUBA3D*) located in the 2q21 region and *kinesin family member 25* (*KIF25*) located in the 6q27 region are involved in microtubule formation. The copy number gain observed at 2q21 was maternally inherited, while the gain seen at 6q27 was paternally inherited. We suspect based on segregation analysis that these duplications, in addition to the potentially pathogenic variant identified in the *FBN1* gene (previously discussed) could be responsible for the skeletal and muscular abnormalities observed in the twins, however in order to confirm this functional studies would need to be performed.

### Cost considerations

When directly comparing the costs of target-capture sequencing to WES, the former is about one fourth lower than that of WES (Table 4). The large target size of WES prohibits high multiplexing capabilities, commonly sequenced with only three samples per lane. This experimental setting results in an average depth of ~50X, while the reduced capture size for of our target panel (4.98Mb) permits extensive pre-capture pooling and increased multiplexing capabilities dramatically lowering the costs. A panel of ~5Mb, and 150bp paired-end sequencing, allows the multiplexing of 16 samples per lane generating an average predicated depth of ~150X, which is still much higher than that usually generated by WES. When directly comparing WES and target capture design using the exact same approach (Roche Nimblegen SeqCap EZ capture, and sequencing using the Illumina HiSeq 2500) the reduced costs are largely driven by increased multiplexing capabilities and the lower costs of the target capture pool (Table 4). Costs associated to library preparation as well as hands on time remain the same.

**Table 4:** Cost comparison of target sequencing panel Einstein_v1 versus Whole Exome Sequencing.

	Einstein _v1	Whole Exome Sequencing
**Capture method**	Roche Nimblegen SeqCap EZ	Roche Nimblegen SeqCap EZ Exome v3.0
**Targeted Region**	4.98Mb	64 Mb
**Number of Probes**	26,046	>2,100,000
**Probe Type**	Biotinylated DNA bait	Biotinylated DNA bait
**Genomic DNA input required**	1ug	1 ug
Number of variants *	4,093 SNVs/758 InDel	6,350SNVs/2064 InDel
**Number of variants MAF <1%**	734 SNVs	1,190 SNVs
**Capture kit, cost per sample**	$37.5	$600
Price sequencing per sample **	$87.5 (16 samples/lane)	$466 (3 samples/lane)
**Cost of library prep**	$200	$200
**Estimated cost**	**$281.25**	**$1,266**

* The number of SNVs/InDels identified was based on samples used in the current analysis (n = 2 for WES and matching target sequencing).

** Based on estimated $1,400/lane 150 bp pair end sequencing on Illumina 2500.

In addition despite the increasing efficacy of pipelines and automated analytical plug-ins being continuously developed, lists of filtered rare variants generated by the adopted pipelines still require manual inspection by trained personnel. When comparing the number of variants identified (vcf files comparison) by the target capture panel (Einstein_v1) vs. WES on the same samples, the target capture approach generates ~40% less (when running the same analytical pipeline). The number of variants that require manual inspection considerably affect the time and the costs required analysis. We estimated, based on our experience, that 8 minutes are required to perform a general analysis of one variant. These include visual inspection using IGV, database, and literature screening to determine whether the variant has been previously reported).

## Discussion

This study highlights the challenges and benefits of developing a multi-disease sequencing assay that could be implemented as a diagnostic tool to assist clinical care in an underserved minority population such as the one that exists in the Bronx. Patients seeking care in depressed areas are often unable to get access to genetic testing offered by commercial laboratories due to high costs of the testing. We selected a cohort of patients to test a target-capture approach, followed by high-throughput sequencing, of exons, exon-intron boundaries and 5’ untranslated regions of 650 known and candidate disease-causing genes related to the cohort of patients selected for this study. The target disease areas included a variety of heterogeneous disorders ranging from known Mendelian-inherited conditions to complex pediatric disorders based on discussions with clinicians from our neighboring hospital. Genome-wide Affymetrix 6.0 SNP/CNV microarrays were performed on the DNA obtained from the same patients to complement target sequencing and assess DNA copy number variations larger than 200 kb. By using both targeted-sequencing and chromosome microarray, our study suggests that an approach based on a large custom sequencing panel combined with CNV analysis is useful as an aid in clinical care. Our target sequencing results exhibited high sensitivity and specificity in terms of accurately identifying previously reported variants proving suitable for testing of the case patients. We could identify a likely disease-associated variant in four patients. These were patients 90.001 and 90.002 with a heterozygous variant in *FBN1* and copy number gains in *TUBA3D* and *KIF25*, patient RB1 with nonsense and splice-site variants in CASQ2, and patient *SS1* with a copy number deletion in *CTTNA3*. In addition based on the sequencing results of our assay we could perform target Sanger Sequencing analysis on some of the patients’ family members pinpointing to a possible pathogenic variant in the offspring.

Lack of obvious disease causing variants in the other samples could be due to their absence in our gene panel, an over all limitation in this type of studies, and therefore requiring a more comprehensive analysis [5]. It is also known that the positive success rates, especially those targeting channelopathies and cardiomyopathies, range from 20–75% [30], indicating that not all genes linked to these disorders have been discovered. Alternatively, variant classification curated by several public variant databases and pathogenicity prediction tools may suggest being benign [25]. However, a position should be interrogated sufficiently and possibly followed up by functional studies in order to rule-out potential false-negative and false-positive results and clarify its true pathogenicity [25,31]. We identified several variants that were detected in both dbSNP and NHLBI but were classified as benign and/or likely pathogenic suggesting the need for more extensive functional work and advancement in pathogenicity prediction tools to better understand the penetrance of previously identified disease-causing variants, especially in a less frequently sequenced minority population. These findings suggest that efforts to make available sequencing tools for an ethnically diverse minority population are an essential step towards a better understanding of variants of unknown significance. Despite the efforts of large sequencing consortiums the number of variants that don’t have an associated MAF and the ones being predicted with contradictory outcomes (tolerated vs. damaging) by various predictor tools is still extremely large. This makes the identification of disease related variants very challenging.

We suggest that custom panels similar to the ones we describe can serve as cost-effective tools that can provide valuable insight to a patient's disease and possible molecular diagnosis. In addition sharing clinical cases and their associated predicted pathogenic variants trough the literature and the dedicated databases is essential to build knowledge in this quickly evolving field. A custom design covering a large number of candidate genes seems to be advantageous for low volume sequencing facilities since this will avoid delays in sample processing due to the need to reach certain multiplexing requirement necessary to contain costs.

Until a large number of sequences of patients from a mixed population, such as the Bronx, is generated and made publicly available, similar to that of the 1000Genomes and NHLBI ESP Projects, it will be challenging to understand the penetrance of previously reported variants among minority and mixed populations. This is likely to be achieved if cost-effective comprehensive panels are made available in clinical settings. Furthermore, sequencing of large cohorts can assist in the discovery of newly pathogenic variants unique to these ethnicities.

## Conclusion

To the best of our knowledge, this study is the first to analyze multiple-diseases and syndromes in a single targeted-sequencing assay. With the approach, we report and discuss the identification of variants in five of the patients from a cohort of individuals selected from the Bronx County in New York. A high throughput, multiplexed target sequencing approach based on a multi-disease design is more cost-efficient than a single, or small panel, candidate gene approach. Therefore clinical centers and hospitals serving low-income neighborhoods may consider a similar approach. A multi-disease design also has the capability to improve detection rates in complex and heterogeneous disorders, ultimately leading to more accurate diagnoses, improved treatment options, and better genetic counseling possibilities. Currently, a multi-disease sequencing panel is available only in research settings. We believe that our study demonstrates the clinical potential that a multiple disease-sequencing panel could provide if implemented in the clinic as a molecular diagnostic tool for the characterization and diagnosis of heterogeneous disorders.

## Supporting Information

S1 Fig CNVs validation using qPCR. From top to bottom: CTNNA3 validation in sample SS1; KIF25 validation in samples TG90’ and TUBA3D validation in samples TG90.
(TIF)

S1 Table Picard stats for WES controls. (XLSX)

S2 Table Complete gene list and associated syndromes. (XLSX)

S3 Table Regions excluded from panel and reason. (XLSX)

S4 Table List of variants used for Sanger sequencing validation and results. (XLSX)

S5 Table Mean depth as function of GC content. (XLSX)
