# Supplementary figures and images for: Development of a Targeted Multi-Disorder High-Throughput Sequencing Assay for the Effective Identification of Disease-Causing Variants
Source: PLoS One. 2015 Jul 27;10(7):e0133742. doi: 10.1371/journal.pone.0133742 (PMC4516357; doi:10.1371/journal.pone.0133742)

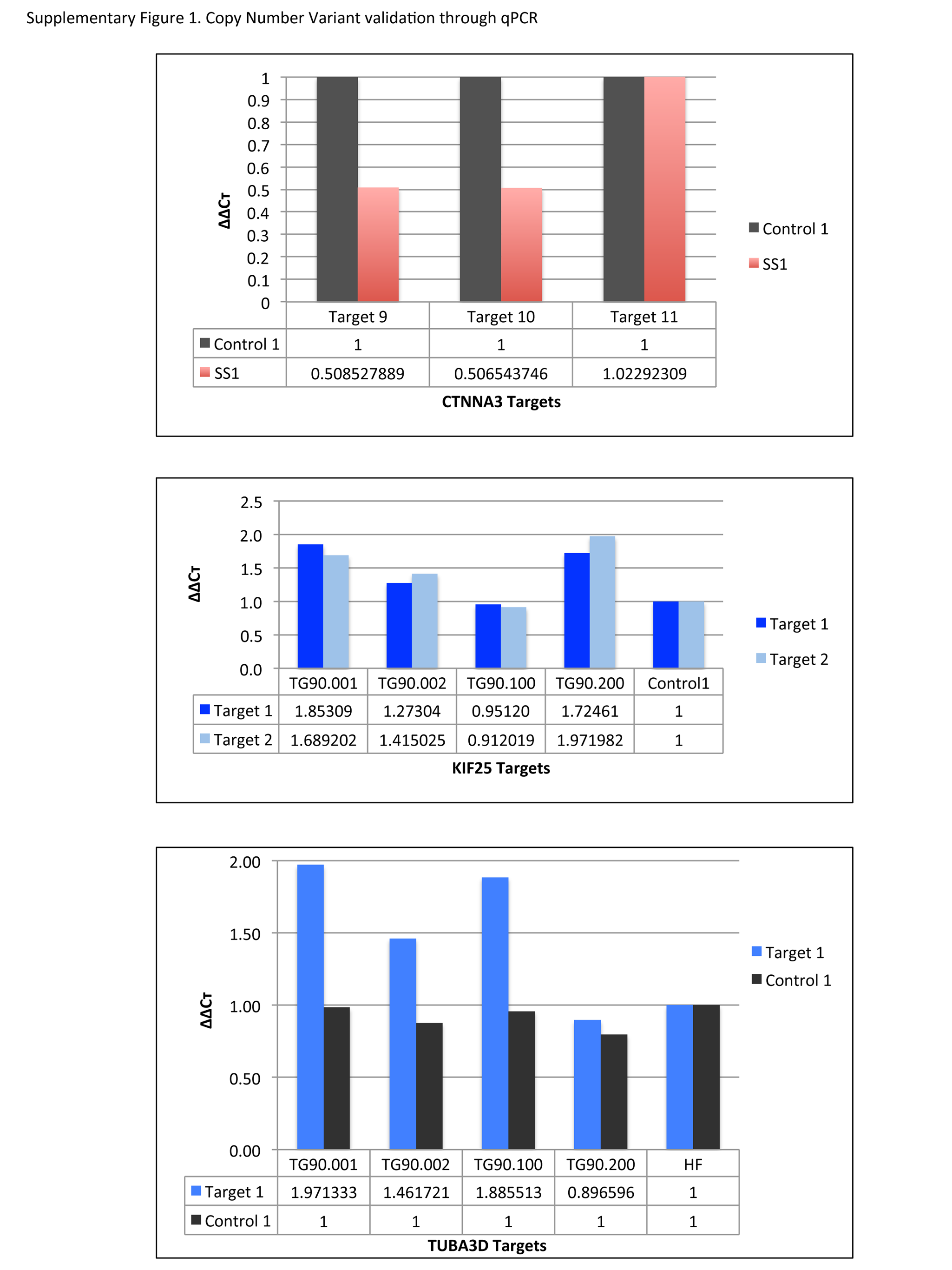

Supplement: S1 Fig — From top to bottom: CTNNA3 validation in sample SS1; KIF25 validation in samples TG90’ and TUBA3D validation in samples TG90. (TIF) [file pone.0133742.s001.tif]
